# An Exophytic Pancreatic Mass Diagnosed as Ganglioneuroma

**DOI:** 10.14309/crj.0000000000001677

**Published:** 2025-04-16

**Authors:** Jinye Liu, Abdulazeez Swaiti, Saeed Graham, Alessandra Martorella, Sha Yi, Kim Geisinger, Zarak Hassan Khan, Kara Regan

**Affiliations:** 1 Department of Internal Medicine, East Carolina University Health Medical Center, Greenville, NC; 2 Department of Internal Medicine, MedStar Georgetown University Hospital, Washington, DC; 3 Department of Pathology, East Carolina University Health Medical Center, Greenville, NC; 4 Department of Gastroenterology, East Carolina University Health Medical Center, Greenville, NC

**Keywords:** pancreatic ganglioneuroma, endoscopic ultrasound, neural crest cell tumors

## Abstract

Ganglioneuromas are rare neuroendocrine tumors affecting around one in a million, mostly young adults. These slow-growing, well-differentiated neuroepithelial tumors originate from the neural crest and involve the sympathetic nervous system. We present an unusual case of a 47-year-old African American man with mildly elevated liver enzymes who was incidentally found to have a suspected pancreatic mass. Endoscopic ultrasound and fine-needle biopsies confirmed the diagnosis of a ganglioneuroma, inseparable and exophytic in the body of the pancreas extending into the tail.

## INTRODUCTION

Ganglioneuromas (GNs) originate from autonomic neural crest cells and usually affect young adults predominantly in the mediastinum, retroperitoneum, adrenal glands, and cervical region.^1^ They are well-circumscribed solid masses with thin pseudocapsules.^2^ Diagnosis involves identifying ganglion cells and specific targeted proteins.

Pancreatic carcinomas are the fourth leading cause of cancer death in the United States with a survival rate of 6%.^3^ Only 20% are resectable at diagnosis.^3^ Pancreatic ganglioneuromas, on the other hand, are extremely rare without any documented incidence or mortality rates, making it difficult to ascertain epidemiological data. We present a rare case of an exophytic pancreatic mass diagnosed as a GN, and we highlight imaging and tests used to evaluate the mass and nearby encased structures, ultimately guiding surgical considerations in a tumor that is not extensively studied.

## CASE REPORT

A 47-year-old African American man with hypothyroidism, obstructive sleep apnea, prior cholecystectomy, and hyperlipidemia presented for routine outpatient annual follow-up with incidentally found mildly elevated liver enzymes. He was asymptomatic, with no history of alcohol, hepatotoxic product use, or relevant personal or family history of cancer. An acute viral hepatitis panel, iron studies, and serum carbohydrate antigen 19-9 were all unremarkable. Right upper quadrant ultrasound demonstrated a hypoechoic 7.2 × 4.9 × 5.4-cm mass in the pancreatic tail. Follow-up computed tomography (CT) discovered a 6.5 × 5.8 × 3.3-cm cystic mass in the pancreatic body without pancreatic ductal dilatation or parenchymal atrophy.

**Figure 1.**
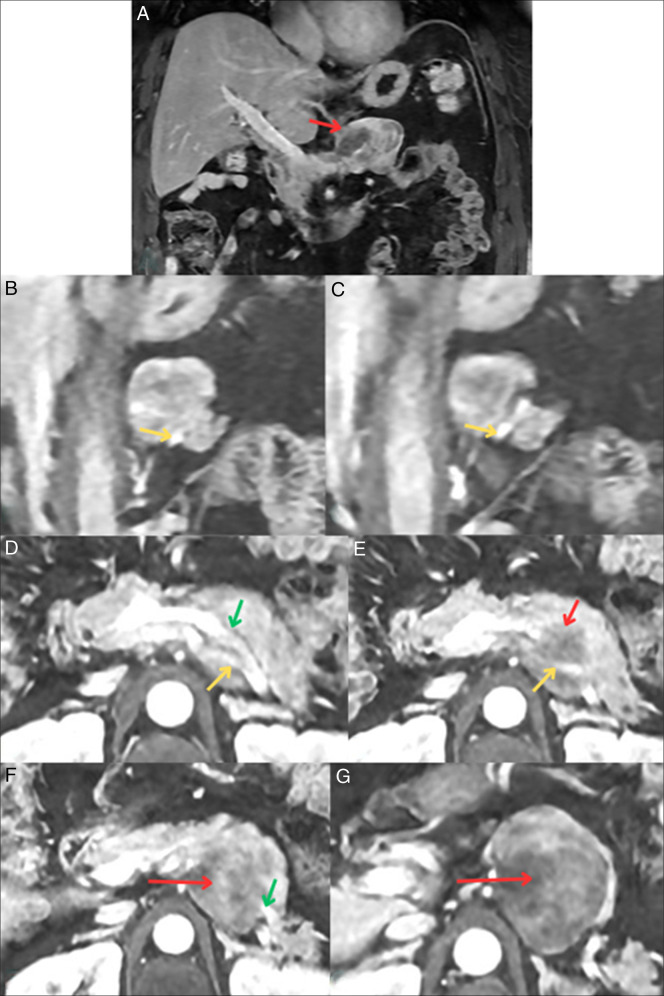
Magnetic resonance imaging abdomen with and without contrast demonstrating coronal views (A–C) of the mass (red arrow) with suggestions toward abutting the splenic vessels: splenic artery (green) and splenic vein (yellow). Axial views (D– G) showing similar findings.

**Figure 2.**
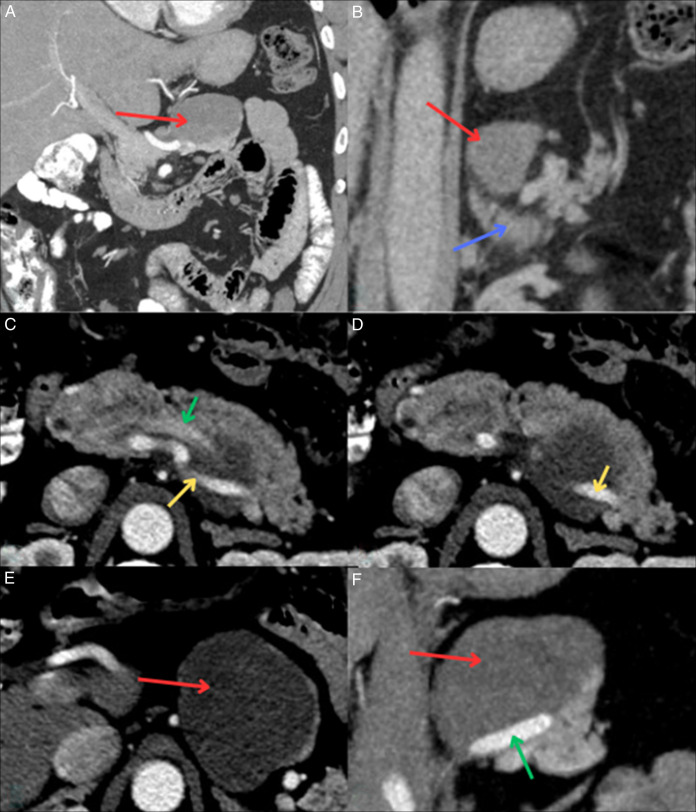
Computed tomography dual pancreas with mass (red arrow) demonstrating no overlap with the left adrenal gland (blue arrow, B) and confirming splenic vessel abutment by the mass on the splenic vein (yellow arrow) and splenic artery (green arrow). A, B, F are coronal views, and C, D, E are axial views. Red arrows also demonstrate mass that is oval and solid in nature with smoother borders, typical of what ganglioneuromas may appear as.

**Figure 3.**
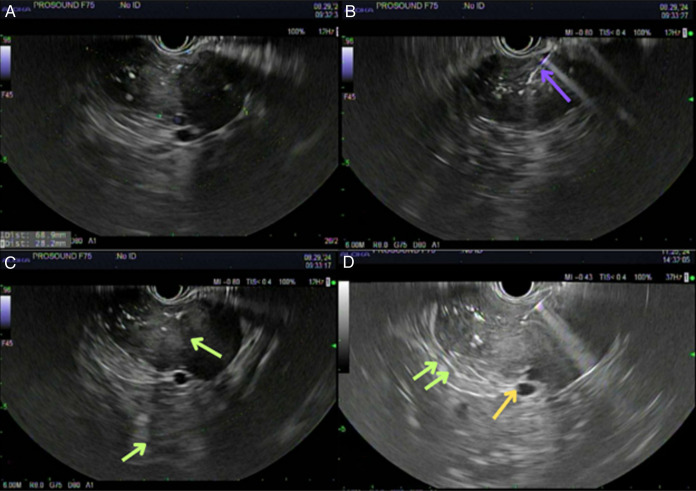
First EUS demonstrating a 68 × 28-mm pancreatic cyst that is anechoic/hypoechoic (A), with purple arrow pointing to fine-needle aspiration biopsy needle (B), and shadowing thought to be small calcium deposits demonstrated by green arrows (C). Second EUS demonstrating same green arrow shadowing as well as abutment of splenic vessels as shown as yellow arrow. EUS, endoscopic ultrasound.

**Figure 4.**
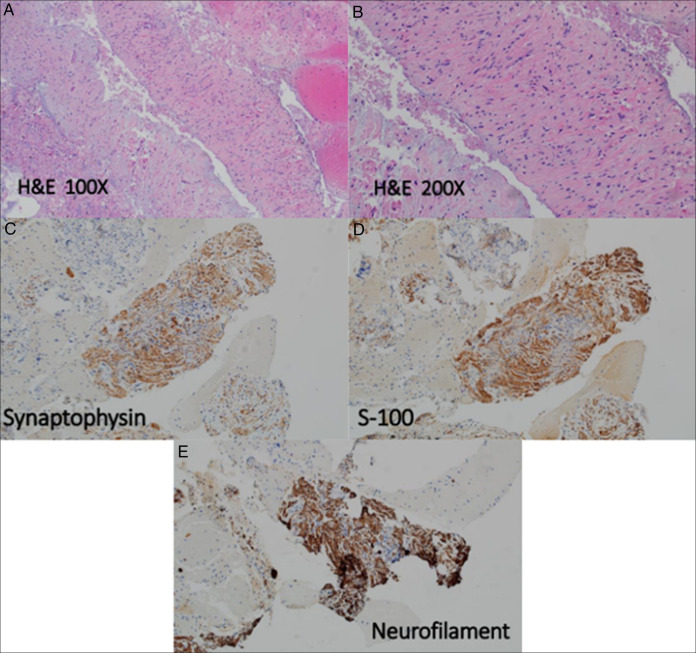
Fragments of large, scattered polyhedral ganglion cells with abundant cytoplasm, large nuclei, and fibrillary stroma with wavy spindle shaped cells and cigar shaped nuclei (A and B). These neoplastic cells are diffusely positive for synaptophysin (C), S-100 (D), and neurofilament (E) and supports the interpretation of a ganglioneuroma. The neoplastic cells are negative for DOG-1, CD117, pancytokeratin, and desmin. H&E, hematoxylin and eosin.

Endoscopic ultrasound with aspiration collected 0.4-mL blood-tinged, thick and viscous fluid and scattered debris. Molecular testing returned as negative; however, gene fusion studies and molecular interpretations were equivocal. MRI confirmed the mass but raised questions about splenic vessel abutment (Figure 1). A subsequent CT dual pancreas confirmed splenic vessel abutment; although the mass did come close to the left adrenal gland, it did not appear that it stemmed from the organ (Figure 2). Repeat endoscopic ultrasound showed a mass measuring 6.8 × 2.8-cm mass in the upper abdomen/perigastric region inseparable from the pancreatic tail (Figure 3). The origin of the mass was indeterminate; however, given its appearance and how it was inseparable and exophytic from the pancreatic body extending into the tail, it maintains a strong possibility that it was pancreatic in origin. Ultimately, through collections of spindle cells and ganglion cells, both of which by immunochemistry tested positive for synaptophysin, S100 protein, and neurofilament stain, biopsy results confirmed a final diagnosis of GN (Figure 4). Although the mass appeared benign without any compressive effects, its large size warranted a thorough pathological assessment. Therefore, a left pancreatectomy and splenectomy (with appropriate vaccinations before surgery) was recommended. The patient was referred to surgery and is still pending surgical evaluation to this day.

## DISCUSSION

GNs are benign rare neoplasms originating from neural crest cells during embryogenesis, and aberrations in their growth signaling pathways can lead to their formation. Rare genetic mutations in rearranged during transfection, anaplastic lymphoma kinase, or PHOX2B pathways may contribute to their formation.^4^ GNs consist of mature ganglion cells and Schwann cells. Diagnosis involves identifying ganglion cells through hematoxylin and eosin staining, and markers like S100 proteins and neuron-specific enolase.^5^ Differential diagnosis includes schwannomas and neurofibromas; however, these do not exhibit ganglion cells. Other differentials include a pancreatic neuroendocrine tumor (PNET), solid pseudopapillary neoplasm, acinar cell carcinomas, and lymphomas. Higher up differentials of PNET were entertained; however, our patient did not exhibit clinical syndromes of hormonal secretion. PNETs also do not have ganglion cells.

GNs range from 1.5 to 20 cm, averaging 6 to 10 cm in diameter at time of diagnosis.^6^ They often grow slowly and are asymptomatic. About 40% arise in the posterior mediastinum, and around 35% in the retroperitoneum, with rarer sites including adrenal glands (∼20%) and cervical spine (∼10%).^7,8^ Two-thirds of cases occur in female patients, while 25% are diagnosed incidentally.^9^ The prognosis of patients at time of diagnosis with stage I, II, and III has a 99.2% survival rate as opposed to stage IV at 65.2%.^10^

The only definitive treatment option for pancreatic cancer is surgical resection. Risk factors may include chronic pancreatitis, smoking, age, diabetes, obesity, family history, and work exposure. Although the pancreas is a retroperitoneal organ, it constitutes a very small percentage of all reported cases of primary retroperitoneal tumors (0.7%–1.6%).^11^ The pancreas is a dense bundle of sympathetic nerve fibers. The hypothesis that dysregulation of these rich, dense bundles of neural networks can lead to growth of GNs. The argument for the rarity of these pancreatic GNs, however, are thought to be due to innervation density, explained by the complex dense system of exocrine and endocrine functions that limits localized proliferation of neural crest cells. Genetic anomalies are not commonly found in pancreatic GNs. Ultimately, they are extremely unusual and rare, with fewer than 10 cases reported.^12^ After review of existing cases in literature, it was discovered that most patients presented with epigastric or left-sided abdominal pain, and 1 case presented as an incidental finding due to a motor vehicle collision; most individuals were female and of early childhood to young adult ages (3-33, however, 1 was 86 years old), and all but the case with the 86-year-old required surgical resection for masses that ranged around 1 to 7 cm in size for curative and diagnostic intent.^12–16^

A systematic approach is essential for diagnosis. Unfortunately, no specific imaging features definitively diagnose GNs. On contrast-enhanced CT, the lesion is normally nonenhanced, but calcifications may be observed. For our patient, a CT indicated a pancreatic mass, prompting further evaluation with MRI, as the thought that parenchymal involvement could be a possibility, and after revealing the mass possibly arising from an adjacent structure, a CT dual pancreas was ordered, suggesting splenic vessel abutment. This advanced imaging uses 2 distinct phases of contrast enhancement: arterial and venous phases to distinguish normal pancreatic tissue from tumors. Hypervascular lesions are identified in the arterial phase and hypovascular ones in the venous phase, each of which help differentiate between normal pancreatic tissue and tumors. It also provides detailed visualization of which blood vessels are invaded by pancreatic cancer, aiding in surgical planning.^17^ The utility of multiple imaging modalities reveal both the extent of the tumor and the organ of origin, but also if there is regional or diffuse invasion, or vascular encasement. Additional imaging, such as 68 Ga-DOTATATE positron emission tomography/CT, may have provided further diagnostic clarity as well. In this scan, a specific tracer is injected and then taken up by somatostatin receptors. Pancreatic GNs may exhibit these receptor expressions; however, uptake pattern can sometimes overlap with other tumors like PNETs, which may confuddle differentials and still lead us to the necessity of histopathological confirmation. Fluorodeoxyglucose-positron emission tomography/CT may also be of utility; however, uptake pattern overlap may still remain. Finally, endoscopic ultrasound with fine-needle aspiration may be used for different indications such as drainage, pain management, and structural assessments, and most importantly, as an official diagnostic tool with biopsy specimens and pathology interpretations.

Currently, no specific management guidelines exist for pancreatic GNs given its rarity, but they generally follow guideline protocols for benign tumors. If the mass appears well-defined and uniform without concerns for features of malignancy, conservative management such as close follow-up with imaging may be appropriate. However, factors such as size and imaging characters influence decision making.

Ultimately, the rarity and commonly nonspecific presentation and imaging findings of pancreatic GNs emphasizes both the importance of combined imaging for holistic care and the necessity of biopsy and surgical removal as the option for definitive diagnosis and treatment, as it is important to differentiate the tumor from malignant neural crest–derived tumors.

## DISCLOSURES

Author contributions: Writing manuscript and literature research: J. Liu, A. Swaiti, S. Graham, A. Martorella, S Yi, K Geisinger. Supervision case and writing manuscript: Z. Khan, K. Regan. J. Liu is the article guarantor.

Financial disclosure: None to report.

Informed consent was obtained for this case report.
